# Improved ICU mortality prediction based on SOFA scores and gastrointestinal parameters

**DOI:** 10.1371/journal.pone.0222599

**Published:** 2019-09-30

**Authors:** Yehudit Aperstein, Lidor Cohen, Itai Bendavid, Jonathan Cohen, Elad Grozovsky, Tammy Rotem, Pierre Singer

**Affiliations:** 1 Department of Industrial Engineering and Management, Afeka Academic College of Engineering, Tel Aviv, Israel; 2 Department of General Intensive Care and Institute for Nutrition Research, Rabin Medical Center, Beilinson Hospital, Petah Tikva, Israel; Vita Salute University of Milan, ITALY

## Abstract

**Background:**

The Sequential Organ Failure Assessment (SOFA) score is commonly used in ICUs around the world, designed to assess the severity of the patient's clinical state based on function/dysfunction of six major organ systems. The goal of this work is to build a computational model to predict mortality based on a series of SOFA scores. In addition, we examined the possibility of improving the prediction by incorporating a new component designed to measure the performance of the gastrointestinal system, added to the other six components.

**Methods:**

In this retrospective study, we used patients’ three latest SOFA scores recorded during an individual ICU stay as input to different machine learning models and ensemble learning models. We added three validated parameters representing gastrointestinal failure. Among others, we used classification models such as Support Vector Machines (SVMs), Neural Networks, Logistic Regression and a penalty function used to increase model robustness in regard to certain extreme cases, which may be found in ICU population. We used the Area under Curve (AUC) performance metric to examine performance.

**Results:**

We found an ensemble model of linear and logistic regression achieves a higher AUC compared related works in past years. After incorporating the gastrointestinal failure score along with the penalty function, our best performing ensemble model resulted in an additional improvement in terms of AUC metrics. We implemented and compared 36 different models that were built using both the information from the SOFA score as well as that of the gastrointestinal system. All compared models have approximately similar and relatively large AUC (between 0.8645 and 0.9146) with the best results are achieved by incorporating the gastrointestinal parameters into the prediction models.

**Conclusions:**

Our findings indicate that gastrointestinal parameters carry significant information as a mortality predictor in addition to the conventional SOFA score. This information improves the predictive power of machine learning models by extending the SOFA to include information related to gastrointestinal organ system. The described method improves mortality prediction by considering the dynamics of the extended SOFA score. Although tested on a limited data set, the results' stability across different models suggests robustness in real-time use.

## Introduction

Patient outcome prediction is invaluable in the ICU setting. The sequential organ failure assessment (SOFA) score is a common scoring tool in ICUs around the world. It is mainly used to assess the severity of a patient's clinical state and to examine the response to a given treatment. The score is calculated daily by summing 13 variables’ values, including vital signs, physical examination and laboratory test results. The score is compiled from six sub-scores, ranging between 0 and 4, assigned to vital systems in the human body, with a higher score indicating increased illness severity. The organ systems assessed for dysfunction are respiration, cardiovascular, liver, renal, coagulation and neurological. Each sub-score represents the level of failure of each of these organ systems. Since its devise in the 1990's [1], originally coined as Sepsis-related Organ Failure Assessment, the correlation between mortality and the SOFA score has been well established [2,3] and the score is considered a major tool for assessing mortality risk in the ICU, alongside other commonly used scoring systems, such as the Acute Physiology and Chronic Health Evaluation and the Simplified Acute Physiology Score (APACHE and SAPS, respectively). The SOFA score is presented in Table 1.

**Table 1:** The sequential organ failure assessment score structure.

System	Parameter, units	0	1	2	3	4
Respiration	PaO_2_ / FiO_2_, mm Hg (kPa)	≥ 400	300–400	200–300	100–200 with respiratory support	< 100 with respiratory support
Coagulation	Platelets x 10^3^/mm^3^	≥ 150	100–150	50–100	20–50	< 20
Liver	Bilirubin, mg/dL (μmol/L)	< 1.2 (20)	1.2–1.9 (20–32)	2.0–5.9 (33–101)	6.0–11.9 (102–204)	> 12.0 (204)
Cardiovascular	Hypotension*	MAP ≥ 70 mmHg	MAP < 70 mm Hg	Dopamine < 5 or Dobutamine (any dose)	Dopamine < 5.1–15 or epinephrine ≤ 0.1 or norepinephrine ≤ 0.1	Dopamine > 15 or epinephrine > 0.1 or norepinephrine > 0.1
Central nervous system	Glasgow Coma Scale score	15	13–14	10–12	6–9	< 6
Kidney	Creatinine, mg/dL (μmol/L) or urine output (ml/d)	< 1.2 (110)	1.2–1.9 (110–170)	2.0–3.4 (171–299)	3.5–4.9 (300–440) < 500	> 5.0 (440) < 200

*Catecholamine doses are given at μg/kg/min for at least 1 hour.

PaO2 = partial pressure of oxygen; Fio2 = fraction of inspired oxygen; MAP = mean arterial pressure.

Data science has been employing computerized models such as Artificial neural networks (ANNs) and logistic regression (LR) for over two decades, trying to improve outcome prediction models [4,5]. Many works of research were dedicated to the SOFA score and its value in predicting mortality, with some results found applicable and potentially beneficial in prognostication and decision making processes. One of the first applications of data science to this problem aimed to assess the correlation between the SOFA score and mortality, with data from 1,449 patients in a multicenter study [5]. This study examined the first SOFA score during a patient’s stay, the maximum score received during a stay and the difference between these two scores. LR was the only model used and it yielded an AUC of 0.847 with the input of maximal sofa score, an AUC of 0.772 with the input of the first day of stay and an AUC of 0.742 with the input of the delta of the scores. In another work [6], LR was used as a wrapper for a custom function, dividing SOFA scores into three categories: a low score between 0 and 6, an intermediate score of 7–8 and a high score of 9 and above. They constructed a feature vector which included the category of the score for each of the first four days in the ICU and presented improved accuracy of mortality prediction. This improvement was associated with the use of several chronological SOFA scores, but still presented heavy processing of data prior to model execution. A systematic review [7] evaluating various SOFA-based models for outcome prediction found SOFA-based models to be as good as SAPS II and only slightly inferior to APACHE II/III when used alone. When used in combination with either of the two models it showed improved performance. However, the review failed to point towards a specific model being superior for accuracy and the need for model improvement remained. Later, other researchers [8] inspected the use of the SOFA score to predict sequences of organ failure during ICU stays, by using Dynamic Bayesian Networks on SOFA’s sub-scores. This study showed that the first organ system failure may be predicated with an accuracy of 71.6%, the second failure with 75.5% and the third with 74.9%.

One of the latest works in the problem domain aimed to predict mortality and a patient’s length of stay (LOS) in the ICU using SOFA-based models and other monitored patient data [9]. This study used a dataset containing 14,480 patients and their SOFA scores and equivalent sub-scores as raw input. The best performing model in this case was the support vector machines with an AUC of 0.82. Another recent work [10] examined the correlations of SOFA scores and ICU mortality within 44 adults which were studied during a period of 8 weeks. SOFA score was determined 24 h postadmission to ICU and subsequently every 48 h for the first 10 days, patients were followed until discharge/death/transfer and later analyzed the collected scores. Strong association (P<0.001) was found between the initial SOFA score, mean and maximum SOFA scores and ICU mortality. This study also indicated strong association between patient outcome and cardiovascular score on day 1 and 3, respiratory score on day 7, and coagulation profile on day 3. Our motivation was to find a lean representation of SOFA which could provide similar or higher accuracy in predicting ICU mortality as these related works.

The Gastrointestinal system has become increasingly recognized as a key player in the development and course of critical illness [11–14]. Gastrointestinal (GI) dysfunction has been shown to be related to worse prognosis [15,16], yet a gastrointestinal dysfunction/failure score is not currently incorporated into the SOFA score as a seventh bodily system. In 2008, Reintam et al. [17] demonstrated that the incorporation of a gastrointestinal score, calculated by a five-grade failure score combining the occurrence of feeding intolerance and intra-abdominal hypertension (IAH) into the SOFA score improved the latter’s predictive power. This study also noted that the mean GI failure score for the first 3 days of ICU admission had a high prognostic value for ICU mortality. A later study in two Egyptian ICUs [18], employing the same GI failure score, examined the predictive power of the SOFA score, GI failure and the combination of the two. It reached similarly positive results. Sun et al. [19] compared a modified GI dysfunction model in patients with severe acute pancreatitis, incorporating clinical, microbiological and radiographic variables and found it superior to Reintam's model in this select patient population. In 2012 the European society of intensive care medicine (ESICM) published the recommendations of an expert panel that included a revised grading system for acute GI dysfunction (AGI grade), mainly based on expert opinions [20]. Its efficacy in predicting worse ICU outcomes in patients with higher AGI grade was later validated in a prospective observational multi-center study [21].

The optimal model for the incorporation of the GI system into the SOFA model has not yet been identified [5]. For a lack of a unified definition as well as other reasons, so far it has not become an accepted part of the SOFA score. Our study examined the potential added value of incorporating GI dysfunction into the SOFA score using a different approach, based on the use of penalty functions in order to correct the SOFA score according to the severity of GI dysfunction. This strategy is considered useful in cases where the suggested GI dysfunction score cannot be calculated from information documented in the ICU or in cases where the outcome contrasts with the SOFA score. In our study we aimed to assess the accuracy of several machine learning models in predicting mortality based on three serial SOFA scores. After examining the models, we then assessed whether the addition of a GI value as a seventh component of the SOFA score might further improve the score's prognostic accuracy.

## Materials and methods

### Patients and setting

This was a retrospective study of a mixed critically ill population, including medical, surgical, trauma and obstetric patients. All patients who were hospitalized between January 1^st^ 2007 and May 1^st^ 2015 in a 16-bed medical-surgical ICU at a tertiary-care, university affiliated hospital were included in the study. Data were drawn from a computerized patient record system (MetaVision ICU ^®^, iMDSoft, Tel Aviv, Israel). The need for providing a dynamic input to the model guided us to select 1,304 patients with three consecutive documented sofa scores. In addition to the SOFA scores each record’s outcome was added to the dataset as a binary variable of mortality or survival of ICU stay, since data concerning long-term survival was not available at the time of the study and will be attainable for future work.

### Ethics approval and consent to participate

The Rabin Medical Center institutional review board has reviewed the study protocol, approved the study and waived the need for informed consent due to the observational nature of the study.

### Computerized analysis

#### Machine learning algorithms and ensemble learning

In this work, we aim to solve a classification problem: to identify whether a patient will survive an ICU stay or not, based on a set of numerical predictors (SOFA scores). We reviewed current literature and looking at commonly used statistical learning methods for diagnosticclassification. Linear Regression [22] Logistic Regression (LR) [22], Support Vector Machines (SVMs) with linear, radial and polynomial kernel function [23][same] and Artificial Neural Networks (ANN) [23].

Logistic Regression: a widely used system for the binary classification problem, i.e. the classification between two options, for example dead or alive. The input may consist of many parameters, measured or calculated, and the output is a value between 0 and 1, that may be interpreted as probability of belonging to one of the two predefined classes.

Linear Regression: this is the simplest tool for regression problems; it may also be used for binary classification although, as opposed to logistic regression, the output values are given in unbounded ranges.

Support Vector Machine (SVM) and Artificial Neural Networks (ANN): it must be noted that both models perform well when data behavior is linear. However, often this is not the case, and other models need to be employed. Both methods can be viewed as black-boxes, meaning there are no transparency and clinical interpretability, potentially restricting the ability to make inferences. SVM produces a binary output, 0 or 1, while ANN may produce either a binary (0 or 1) or a probability (between 0 and 1) output. While interpretation of these models is limited, the prediction accuracy may be higher when the data behavior is not linear.

A more detailed description of these models appears in a supplement to this article (S1 File).

The next step was to examine combinations of multiple algorithms. The rationale was that different algorithms make different assumptions about the data, so they define different classifiers. These classifiers work in parallel and their outputs can be fused to produce a compound, potentially more accurate classifier.

#### Incorporating the gastrointestinal system

We defined a new score, based on the SOFA score, with the addition of a GI failure score. Patients were assessed for GI dysfunction using the scoring system devised by Reintam et al. [17], assigning a value between 0 and 4. This tool is detailed in Table 2. The values were derived from patient records of nursing input regarding vomiting, and bowel movements. Resting Energy Expenditure (REE) was derived from measurements using indirect calorimetry or, in its absence, assessed using the Faisy-Fagon equation [24]. Using the aforementioned computerized models, a later effort towards identifying false positive results (i.e., patients whom were not predicted to survive) was performed and then the seventh parameter, that of gastrointestinal dysfunction, was added to examine whether its addition increased the precision of the scoring system. Data was scaled into a five-grade severity scale in order to fit the structure with that of the SOFA score (each system score ranges from 0 to 4). This scaling is presented in Table 3.

**Table 2:** Gastrointestinal failure score, adapted from Reintam et al. [17].

Score	Definition
0	Normal GI function
1	Enteral feeding <50% of calculated needs or no feeding 3 days after abdominal surgery
2	Food intolerance (enteral feeding not applicable due to high gastric aspirate volume, vomiting, bowel distension, or severe diarrhea) or IAH
3	Food intolerance and IAH
4	Abdominal compartment syndrome

GI: Gastrointestinal; IAH: intra-abdominal hypertension.

**Table 3:** Score scaling on all three variables.

Scaled score	0	1	2	3	4
REE daily balance	less than -500	between -500 and -1000 or between 0 and +500	Between -2000 and -1000 or between +500 and +1000	between -2000 and -3000 or between +1000 and +2000	Less than -3000 or more than +2000
Gastric residual volume, vomiting	small amount (up to 150 ml)	medium amount (150–500 ml)	large amount (over 500 ml), Vomiting,	-	bloody vomiting, fecal vomiting,
Bowel movements	formed (may be of varying quantity)	soft stools, small quantity diarrhea	fecal blocks, stool to ileostomy/colostomy, large diarrhea	requiring bowel management system, no bowel movement, small quantity melena	requiring rectal tube, large quantity melena, frank hematochezia

The proposed new GI dysfunction assessment tool evaluated in our study. We assigned a SOFA-style scoring between 0 and 4 for each of the three parameters except Gastric dysfunction which did not include a score of 3. REE balance was defined as the difference between the caloric target and the amount of calories actually administered. All data was described according to the pre-existing parameters in our patient record system.

REE: resting energy expenditure

#### Penalty function & descriptive regression trees

Selecting the right gastrointestinal variables was not a trivial task, since the predictive power and relevancy of each of the gastrointestinal variables was unknown at this point. We used regression trees as a means of describing the relationship between SOFA scores, gastrointestinal parameters and outcomes (mortality) for each patient. The aim was to correct the existing SOFA score so by adding gastrointestinal dysfunction values. This process is described in detail in S2 File.

#### Comparison between models

A receiver operating characteristic curve (ROC) curve was built, plotting the true positive against the true negative rates at various threshold settings. The area under the curve (AUC), which is represents the probability for a 'positive' occurrence, was plotted using this formula:

$$AUC=\int_{\infty }^{-\infty }TPR(T)FPR'(T)dT$$

The model with the maximal AUC was considered the most favorable. In addition to AUC, we also compared sensitivity, specificity, accuracy, negative predictive value (NPV) and positive predictive value (PPV), all of which are common performance indicators for comparison of predictive models.

## Results

The case records of 4,500 patients were included in our analysis. For the first part of modeling we looked at certain classification algorithms (ANN, SVM, etc.) independently in order to select the best model from each model type. We selected the best performing model from each group. The fusion of logistic and linear regression provided the best results (AUC of 0.9113). We inspected the performance of SVM with three different kernels: linear, radial and polynomial, and selected the best model with 8-fold-cross validation. This process is further detailed in S1 File. Table 4 presents the performance of each SVM model trained with a different kernel, while the best performance was achieved with the polynomial kernel.

**Table 4:** Support Vector Machines (SVMs) results.

	Linear SVM	Radial SVM	Polynomial SVM
Area under Curve (AUC)	0.9061	0.8825	0.9066
Accuracy	0.8323	0.8291	0.8766
Sensitivity	0.6632	0.6526	0.6316
Specificity	0.9050	0.9050	0.9050
FPR	0.0950	0.0950	0.0950

The results of SVM methods using different kernel functions are presented. As the highest AUC was achieved using a polynomial kernel function, this method was assessed to be the superior SVM and only it was used later for comparison with the other models. SVM: Support Vector Machine; FPR: False Positive Rate

**Fig 1:**
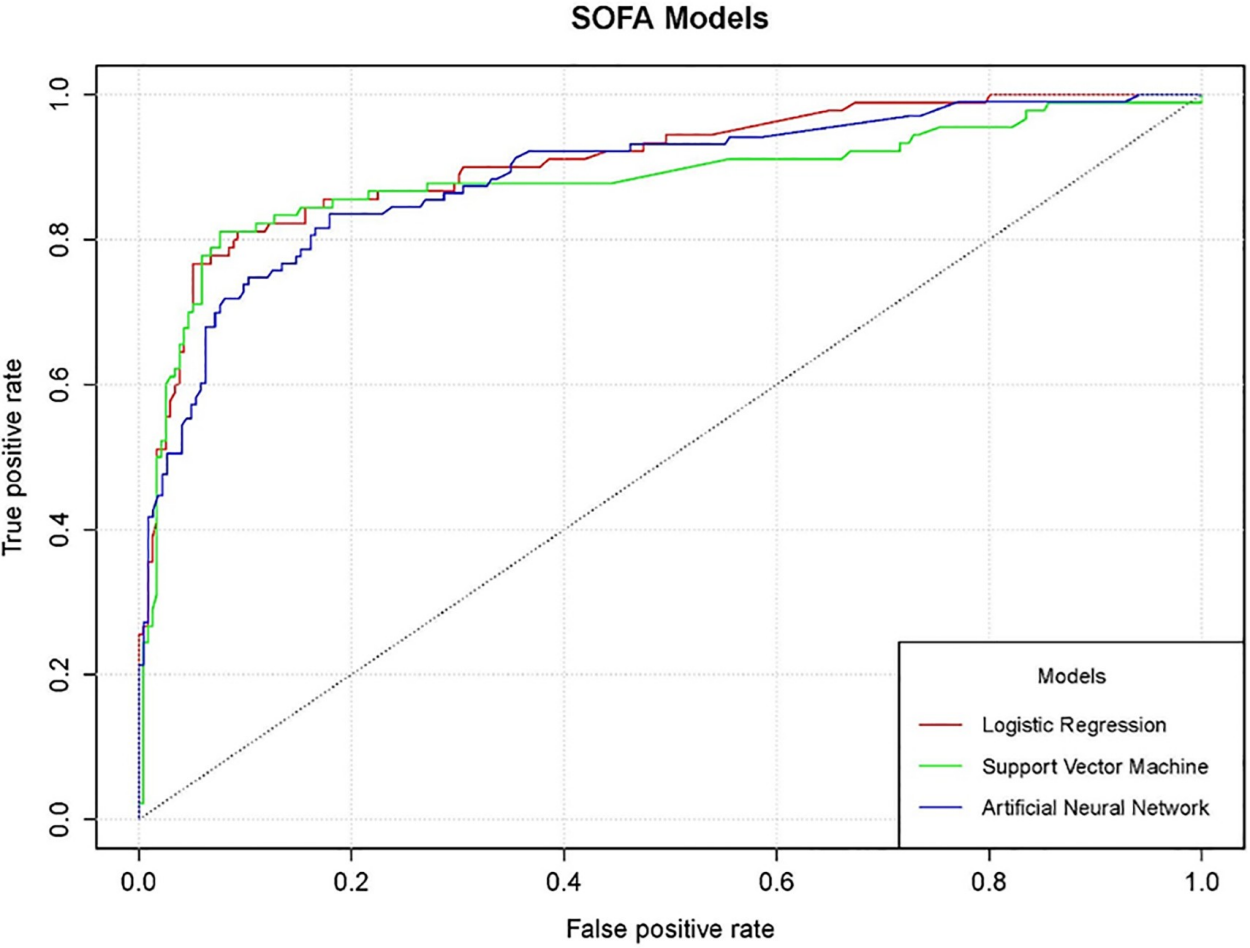
A comparison of classifiers on ROC curve. The Received-Operator Curves (ROCs) of three different classifiers are presented. All three methods (logistic regression, SVM with a polynomial kernel and ANN) produced similar curves, all above 0.9 which is considered highly accurate for classification, with only minute differences between them.

After the Best SVM model was selected, we compared it with other built models such as the ANN and the logistic regression model. For a graphical comparison of models, we used the ROC curve to asses which model performs best on the available data. Fig 1 presents the ROC curve for each model plotted together for best comparison.

As the performance of the different classifiers was similar according to Receiver-Operator Curves, we decided to employ ensembles of the different models to further improve diagnostic ability. We constructed the following ensembles with combinations of the aforementioned models. Table 5 displays the performance of all classifiers and ensemble classifiers, where it is evident that the best AUC is achieved with the ensemble of logistic and linear regression. This finding is somewhat intuitive given the ordinal nature of the input scores we used (both SOFA and gastrointestinal scores are on an ordinal scale).

**Table 5:** Full results comparison (without GI parameter).

Model	Area under Curve (AUC)
ANN	0.8875
SVM (Polynomial kernel)	0.9066
Linear Regression	0.9070
Logistic Regression	0.9070
Ensemble 1: ANN + Linear Regression	0.9101
Ensemble 2: Logistic + Linear Regression	0.9113
Ensemble 3: ANN + SVM + Linear Regression	0.9072
Ensemble 4: ANN + SVM + Linear + Logistic Regression	0.9081

A comparison of the performance of the different models as well as ensemble methods, i.e. combinations of single methods, shows that the ensemble of logistic and linear regression produced the highest AUC. GI: gastrointestinal. AUC: area under the curve. ANN: artificial neural networks. SVM: support vector machine.

After finding the best performing ensemble, we looked at improving results with the addition of the GI dysfunction score. We used a penalty function to correct the SOFA score when the actual outcome did not accord with the score.

At this point, using the 3 latest SOFA scores of a patient, we reached a level of overall accuracy which was higher than past finding in the literature, but still there were misclassified cases which we wanted to minimize. These cases were in fact false positives (patients which survived their ICU stay, but the model classified them as not likely to survive the stay). It became evident from the data that the majority of these cases were such that the last 3 SOFA scores were rising, implying a worsening in patient condition, even though that patient survived. We hoped the gastrointestinal system could shed some light on these errors, by explaining the survival of these patients by their nutritional condition, therefore improving model performance. We looked at the three latest SOFA scores only, three latest SOFA scores with Zb value (SOFA + Zb) and three latest SOFA scores with gastrointestinal scores and Zb values. We evaluated these inputs on our ensemble models and found the combination of the latest three SOFA scores, the addition of the GI failure tool as well as the penalty function (Zb) to yield the best results (AUC = 0.9146). This performance analysis is presented in Table 6.

**Table 6:** Performance of all inspected inputs (with GIF).

# models	ANN	Poly SVM	Linear Reg.	Logistic Reg.	SOFA	SOFA + Zb	SOFA + Gastrointestinal with Zb
**1**	✓				0.8875	0.9077	0.9024
**1**		✓			0.9066	0.9076	0.9146
**1**			✓		0.9070	0.9087	0.9036
**1**				✓	0.9070	0.8855	0.8645
**2**	✓		✓		0.9101	0.8960	0.9033
**2**			✓	✓	0.9113	0.9096	0.9020
**2**		✓	✓		0.9102	0.9093	0.9080
**3**	✓	✓	✓		0.9072	0.9098	0.9100
**4**	✓	✓	✓	✓	0.9081	0.9086	0.9046

A comparison of the inspected models, single as well as ensembles, before and after the addition of a GI dysfunction tool. It reveals better predictive capabilities for the addition of the GI dysfunction score to the SOFA score with a penalty function (Zb). # MODELS: 1 signifies a single model, 2 to 4 signify ensembles. GIF: gastrointestinal failure; SVM: Support Vector Machine; ANN: artificial neural networks; SOFA: Sequential organ failure assessment; Reg.: regression.

## Discussion

There is an ongoing effort to improve prediction models for patient outcome in the ICU. In this study we tested the efficacy of using a patient’s latest SOFA scores to represent the change in condition throughout ICU stay for the purpose of predicting ICU mortality. We first examined the ability of the SOFA score to predict mortality on the using the data from our ICU. The need to use sub-scores dictates larger input vectors[9], thus in this work we examined new ways to achieve this level of accuracy with more compact inputs. Using several machine learning algorithms showed good performance of the SOFA score with an AUC mostly above 0.9. We then assessed several ensemble methods and found the combination of logistic and linear regression to slightly improve prediction. Furthermore, since so many models and methodologies were used, examining the different models we observed a range of performance in accuracy, showing a relatively tight interval between 0.8875 and 0.9113. This narrow interval, despite using four different algorithms, ensembles and input combinations, indicates solid results where accuracy is not expected to decline drastically when further tested on new data, possibly from mixed center populations (i.e., patients from other hospitals/countries). The next step was to incorporate a GI failure score with the SOFA score to further improve prediction accuracy. We used descriptive decision trees to discover GI parameters that may be able to reduce prediction error of classifiers based solely on SOFA. In the aforementioned study by Reintam et al. [17], a GI dysfunction score was developed in an effort to further improve the performance of the SOFA score; however, the results were equivocal [16]: although the number of GI symptoms was significantly higher in non-survivors, no symptom could be used as an independent predictor for mortality. Furthermore, the incorporation of the combination of SOFA and GI failure scale to this new heterogenic population failed to improve performance. The final conclusion drawn from these past studies was that a new approach to the problem was required.

It seems that a few obstacles prohibit the GI system's incorporation into severity scoring systems, including the wide diversity of gastrointestinal disorder clinical manifestations in the ICU [25], a lack of an accepted definition for GI failure [26], lacking validation of laboratory markers, mainly citrulline [27], and the scarcity of strong-level evidence. Feeding intolerance, an important manifestation and defining factor for GI failure, is by itself not yet well defined [28], as it may be based solely on GRV measurements, amount of enteral nutrition delivered or GI symptom lists. Understanding of the intricate interrelation between acute GI dysfunction, feeding intolerance and intraabdominal hypertension and their wide areas of overlap is still evolving [29].

We devised a completely new approach for the incorporation of the GI abnormalities into prognostic methods. Our machine learning prediction model combines integrated gastrointestinal disturbances with well-established organ failure severity score. The model significantly improved the prediction capabilities of the standard SOFA score. Moreover, the model analyzes the dynamics of change in these parameters over time, making it a dynamic score (i.e., adding the important element of time). The time series approach allows for a significant improvement in mortality risk prediction compared to a single SOFA score reading. Our research shows that our approach allows the design of a prediction model with improved prediction accuracy of ICU mortality risk, potentially advancing towards the addition of GI component into the SOFA score, thus improving its predictive abilities.

## Conclusions

Our models of data analysis yielded strong evidence for the accuracy of the SOFA-based scoring system. When incorporating the time element by looking at three consecutive SOFA scores and adding a seventh we demonstrated a yet more accurate predictive ability of the model. We believe it represents a step towards a call for the inclusion of the GI system in SOFA-based scoring systems and helps bridge the evidence gap in this field.

## Supporting information

S1 File Machine learning algorithms. (DOCX)

S2 File Penalty functions and descriptive regression trees. (DOCX)

## Abbreviations

SOFA: sequential organ failure assessment
ICU: intensive care unit
APACHE: acute physiology and chronic health
SAPS: simplified acute physiology score
GI: gastrointestinal
IAH: intra-abdominal hypertension
REE: resting energy expenditure
ROC: receiver operating characteristic curve
AUC: area under the curve
LR: logistic regression
SVM: support vector machines
ANN: artificial neural networks
