# Supplementary material for: Improved ICU mortality prediction based on SOFA scores and gastrointestinal parameters
Source: PLoS One. 2019 Sep 30;14(9):e0222599. doi: 10.1371/journal.pone.0222599 (PMC6768479; doi:10.1371/journal.pone.0222599)
Supplement: S1 File — (DOCX) [file pone.0222599.s001.docx]

**Supplement 1: Machine learning algorithms**

All models were configured using R and the CART package with the relevant dataset and model selection parametrization was made using 8-fold cross-validation.

**Support Vector Machines (SVMs)**

This classification model is commonly utilized for medical applications. The goal is to find a hyperplane of the form $w^{T}x+b=0$which will provide the best separation between two classes of examples in the space. The best hyperplane is determined by the widest possible margins which separate it from the closest examples of both classes. Labels of classes are denoted as *y={-1,1}* and the decision function is :

$$f\left(x_{i}\right)=sign\left(w^{T}x_{i}+b\right)$$

so each $x_{i}$ which fulfills $w^{T}x+b>0$ will be classified as 1, and those which fulfill $w^{T}x+b<0$will be classified as -1. In order to produce a probability output in the range [0,1], we will pass SVM’s output to a sigmoid function. In some cases, a linear hyperplane to separate the two classes does not exist, so a kernel function is used. A kernel function is a mathematical manipulation which maps the problem characteristics in the plane to a higher dimension in which the required hyperplane can be found. The input $x_{i}$ is replaced be a kernel function$\Phi\left(x_{i}\right)$*:*

$$\begin{matrix} \mathop{\mathrm{argmin}}\limits_{w,b} \left(\frac{1}{2}\left\|w\right\|^{2}\right) \\ s.t. y_{i}\left(w^{T}\Phi\left(x_{i}\right)+b\right)\geq1 \forall\left(x_{i},y_{i}\right) \end{matrix}$$

Two different kernels were used in this study. The polynomial basis function is of the form:

$$\Phi\left(x_{i},x_{j}\right)=\left(x_{i}^{T}\cdot x_{j}+c\right)^{d}$$

A radial basis function is of the form:

$$\Phi\left(x_{i},x_{j}\right)=e^{-\frac{\left\|x_{i}-x_{j}\right\|^{2}}{2\sigma^{2}}}$$

When using models which return a probability, such as SVMs, the chance an example belongs to a certain target class, such as SVMs a decision must be made to determine the classification threshold, i.e. the minimal output probability which will determine association to output classes, this is in fact a transformation between regression and classification. This threshold is the probability which will minimize the difference between accuracy and False Positive Rate (FPR) when performing k-fold cross-validation.

**Artificial Neural Networks (ANN)**

Artificial Neural Networks (ANN) is a multilayered mathematical representation of a learning network which maps the correlation between inputs and outputs by backtracking to evaluate and minimize errors. This network contains neurons and arcs which comprise the net’s architecture, which can be generally described as so:

$$y\left(k\right)=F\left(\sum_{i=0}^{m} w_{i}\left(k\right)\cdot x_{i}\left(k\right)+b\right)$$

where:

$x_{i}(k)$ - input of the $k^{th}$ neuron where$i=1,...,m$.

$w_{i}(k)$- Value of correlation between the $k^{th}$ and $k-1^{th}$ neurons.

*F-* Propagation function, for classification usually a sigmoid function.

*b* - bias of said neuron.

y(k) – output of $k^{th}$neuron.

New examples are then run through the net from input neurons to outputs in a process called forward propagation.

Best practice usually calls for selecting a number of hidden layers that is equal to the amount of input features minus one, which in this case proved compatible with best performance. From inspecting accuracy as a function of 1 to 19 hidden layers we found that two hidden layers fits the optimal model. Thresholds between 0.05 and 0.95 with 0.05 increments were inspected as well and the optimal threshold of 0.45 was chosen.

**Logistic Regression**

Logistic regression is a common tool for medical data analysis, including mortality or morbidity outcomes prediction. It is common to use it as a benchmark with other more advanced machine learning models. LR builds a function which describes the correlation between input and output variables, the function’s output is forwarded as input to a sigmoid function which assures the overall output is a probability.

$$\begin{matrix} sigmoid:(-\infty,\infty)\to[0,1] \\ P\left(C_{1}/X\right)=sigmoid\left(w^{T}x+w_{0}\right)=\frac{1}{1+e^{-(w^{T}x+w_{0})}} \end{matrix}$$

**Linear Regression**

Given a variable y and a number of variables X1, ..., Xp that may be related to y, linear regression analysis can be applied to quantify the strength of the relationship between y and the Xj, to assess which Xj may have no relationship with y at all, and to identify which subsets of the Xj contain redundant information about y. The linear regression model reached an AUC of 0.9070, equal to that of logistic regression, with the following weights:

$$\begin{matrix} p(Y=Mortality|SOFA_{t-2},SOFA_{t-1},SOFA_{t})= \\ -0.00593\cdot SOFA_{t-2}+0.004454\cdot SOFA_{t-1}+0.072443\cdot SOFA_{t}-0.022052 \end{matrix}$$
