# Supplementary material for: Improved ICU mortality prediction based on SOFA scores and gastrointestinal parameters
Source: PLoS One. 2019 Sep 30;14(9):e0222599. doi: 10.1371/journal.pone.0222599 (PMC6768479; doi:10.1371/journal.pone.0222599)
Supplement: S2 File — (DOCX) [file pone.0222599.s002.docx]

**Supplement 2- Penalty Functions and Descriptive Regression Trees**

Regression trees are machine-learning methods for constructing prediction models from data. The models are obtained by recursively partitioning the data space and fitting a simple prediction model within each partition. Regression trees are for dependent variables that take continuous or ordered discrete values, with prediction error typically measured by the squared difference between the observed and predicted values^1^

To these means, we constructed the following penalty function $Z_{b}$which will be added to each SOFA score and will represent the corresponding gastrointestinal condition. The following function:

$$Z_{b}=q^{n}\cdot a , a\in\left[-2,2\right]$$

where:

$a$- a parameter indicating the relationship between the predicted outcome of the best performing model so far (ensemble of logistic and linear regression) and the actual outcomes as shown in table 1.

$q$- a constant which represents the uncertainty present when predicting in real time, to model the fact that the actual outcome of the patient is unknown at this time. We used a default of 0.9.

$n$- a time factor representing the origin of the SOFA score (a value of 2 for the first of the three, 1 for the middle and 0 for last score).

Table 1: Setting values of parameter a using confusion matrix

| a | Predicted Outcome | Actual outcome | confusion matrix |
| --- | --- | --- | --- |
| 2 | Survival | Mortality | False Positive |
| 1 | Mortality | Mortality | True Positive |
| -1 | Survival | Survival | True negative |
| -2 | Mortality | Survival | False Negative |

Our goal at this point was to train a regression tree which will aim to estimate the value of $Z_{b}$using the following available input: each SOFA, REE, vomitus and bowel movement of each individual ICU stay. The tree model will help us determine in which cases we should add a correction to the SOFA score which takes into account the gastrointestinal system. The regression tree which was built as seen in figure 1.

Figure 1: Regression tree to describe $Z_{b}$ values.


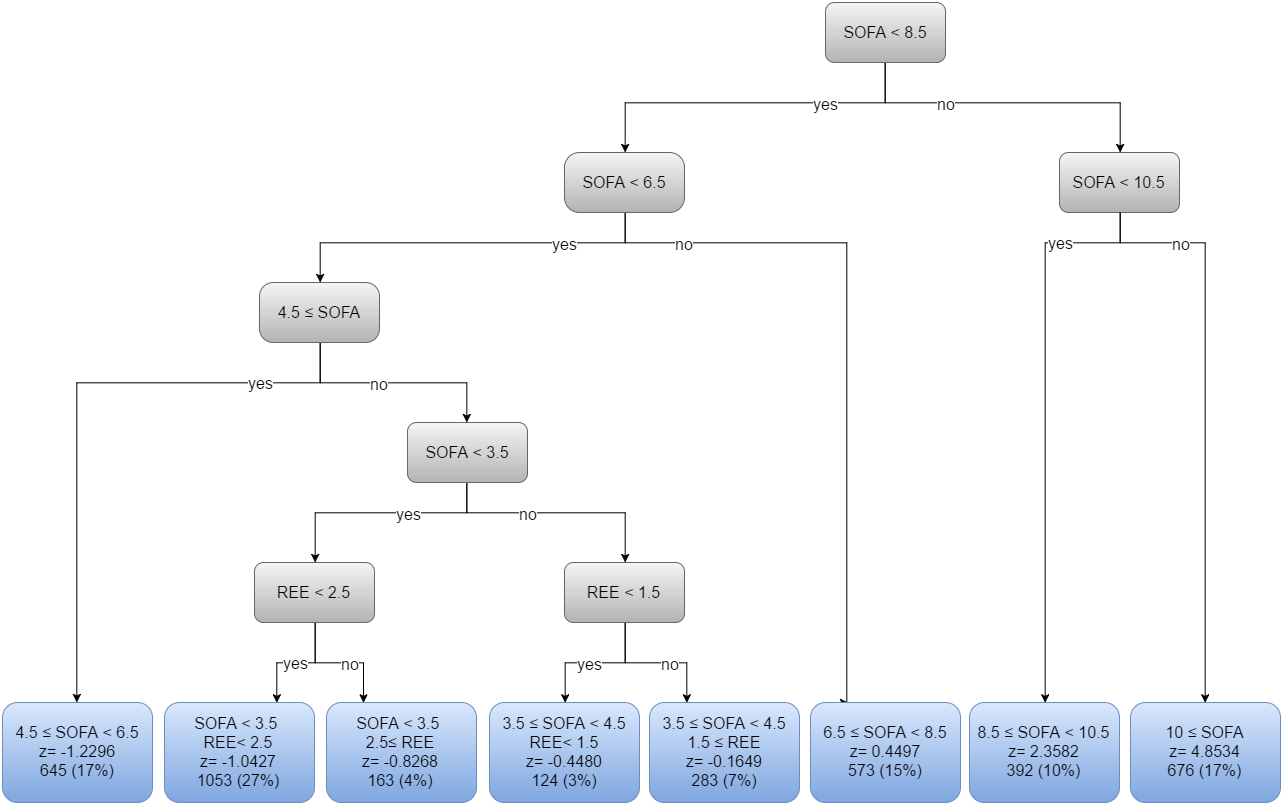


The rule set which is derived from the tree gives us the following penalty values, which will be used as correction to the SOFA scores associated with a patient. This correction helps make the SOFA score better correlated with mortality. Table 2 details the number of cases in the training set which will be fixed, each with its own $Z_{b}$ value.

| Table 2: Rule set for the regression tree – penalty function- $Z_{b}$ | | | |
| --- | --- | --- | --- |
| **Node** | **Rule** | $Z_{b}$ **value** | **number of cases in training set** |
| 1 | 4.5 ≤ SOFA < 6.5 | -1.229612403 | 645 (17%) |
| 2 | SOFA < 3.5 & REE< 2.5 | -1.042756508 | 1053 (27%) |
| 3 | SOFA < 3.5 & 2.5 ≤ REE | -0.826802842 | 163 (4%) |
| 4 | 3.5 ≤ SOFA < 4.5 & REE< 1.5 | -0.448058968 | 124 (3%) |
| 5 | 3.5 ≤ SOFA < 4.5 & 1.5 ≤ REE | -0.164881013 | 283 (7%) |
| 6 | 6.5 ≤ SOFA < 8.5 | 0.449720768 | 573 (15%) |
| 7 | 8.5 ≤ SOFA < 10.5 | 2.358214286 | 392 (10%) |
| 8 | 10 ≤ SOFA | 4.853402367 | 676 (17%) |

Finally, after fixing the SOFA scores, we present the following vectors as input to our ensemble models.

$$\left[\begin{matrix} \begin{matrix} SOFA_{t-2}, & Bowel_{t-2}, & Vomit_{t-2}, & \begin{matrix} REE_{t-2}, & Zb_{t-2} \end{matrix} \end{matrix} \\ \begin{matrix} SOFA_{t-1}, & Bowel_{t-1}, & Vomit_{t-1}, & \begin{matrix} REE_{t-1}, & Zb_{t-1} \end{matrix} \end{matrix} \\ \begin{matrix} SOFA_{t}, & Bowel_{t}, & Vomit_{t}, & \begin{matrix} REE_{t}, & Zb_{t} \end{matrix} \end{matrix} \end{matrix}\right]$$

Thus, the input per patient compiled the three latest SOFA scores, REE, Vomitus, bowel movement and Zb values, used to predict ICU stay morality.

Reference:

1. Loh WY. Classification and regression trees. WIREs Data Mining Knowl Discov 2011; 1:114-23.
